# Supplementary material for: Hydrogenase-based oxidative biocatalysis without oxygen
Source: Nat Commun. 2023 May 31;14:2693. doi: 10.1038/s41467-023-38227-9 (PMC10232426; doi:10.1038/s41467-023-38227-9)
Supplement: Supplementary file 1 — Supplementary Information [file 41467_2023_38227_MOESM1_ESM.pdf]

Supplementary Information

Hydrogenase-based Oxidative Biocatalysis without Oxygen

Ammar Al-Shameri[1], Dominik L. Siebert[1], Samuel Sutiono[1], Lars Lauterbach[2] & Volker Sieber [1, 3,4,5]

*

[1]Chair of Chemistry of Biogenic Resources, Campus Straubing for Biotechnology and Sustainability, Technical University of Munich, Schulgasse 16, 94315 Straubing, Germany.

[2]RWTH Universität Aachen, Institute of Applied Microbiology, Worringerweg 1, 52074 Aachen, Germany.

[3]Catalytic Research Center, Technical University of Munich, Ernst-Otto-Fischer-Straße 1, 85748 Garching, Germany.

[4]SynBiofoundry@TUM, Technical University of Munich, Schulgasse 16, 94315 Straubing, Germany.

[5]School of Chemistry and Molecular Biosciences, The University of Queensland, St. Lucia, Queensland 4072, Australia.

* Corresponding Author: V. Sieber, E-mail: sieber@tum.de

Contents

1. Supplementary Results and discussion 3

1.1. Monitoring the NADH oxidation activity. 3

1.2. NADH oxidation at different NADH and FMN content 4

1.3. Conversion of D-xylose to D-xylonate using both NAD^+ regenerations systems 5

1.3.1. Eliminating the bias- pH, buffer, and temperature screening 5

1.3.2. Influence of O_2, buffer capacity, and FMN 7

1.4. Comparison between the open and closed system 8

1.5. The conversion of D-xylose to α-ketoglutarate overtime 9

1.6. Comparison between SH and NOX in a stirred setup 10

1.7. H_2 production 11

1.8. Diffusion of H_2 from the aqueous phase 13

1.9. Heterologous production of SH in *E. coli* 14

1.10. Scaling up. 15

2. Supplementary References 16

1. Supplementary Results and discussion

1.1. Monitoring the NADH oxidation activity.

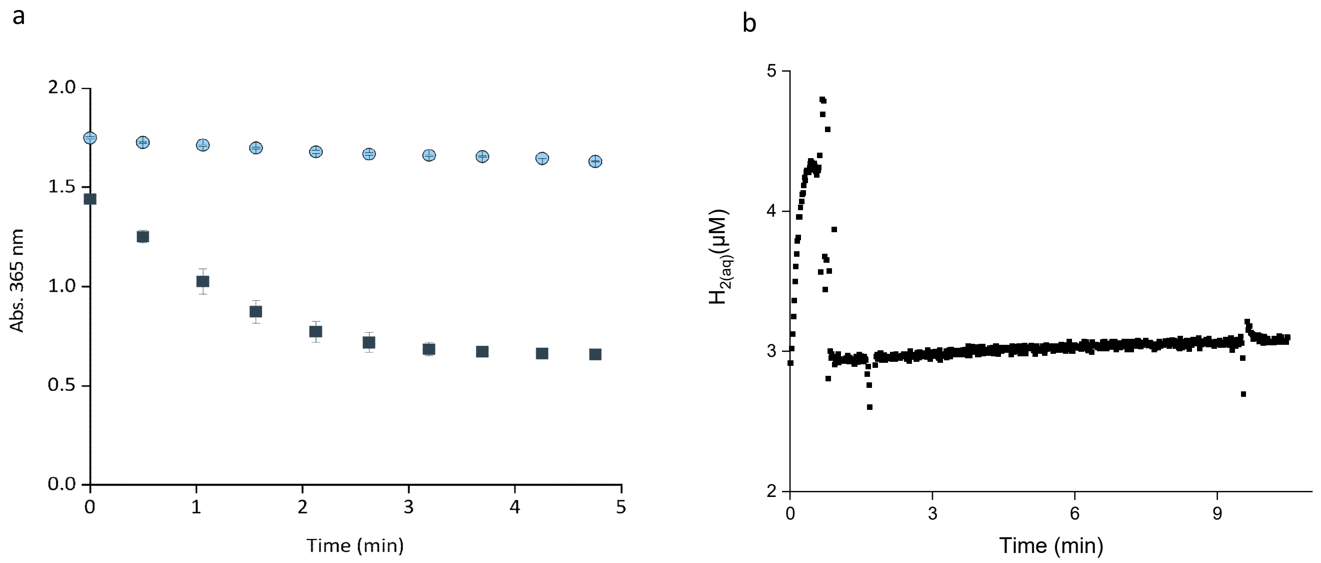

Supplementary Figure1: The SH-mediated oxidation of NADH in the absence and presence of FMN (a), H_2 formation during SH-mediated oxidation of NADH (b).
15 µM of SH was added to Tris-HCl buffer pH 8 containing 1 mM NADH. The NADH oxidation was followed at 365 nm at 22 °C. Blue circles: without FMN. Black rectangular: with 0.1 mM FMN. Each reaction with the same conditions was conducted in independent replicates (n = 3, biologically independent). The data are depicted as mean values with error bars as SD (a). The H_2 concentration during the NADH oxidation was monitored using a microsensor in solution after adding 4 µM of SH (b).

1.2. NADH oxidation at different NADH and FMN content

Supplementary Table 1: The relative NADH oxidation activity of SH at different NADH and FMN concentrations.
The activity test was performed in a microplate in Tris-HCl buffer pH 8 at 22 °C with 15 µM SH. NADH oxidation was followed at 365 nm using the plate reader from Biotek Epoch 2 (Agilent). Data were evaluated using the software Gen5 (Agilent). The grayscale indicates relative activity from 5 % (white) to - 100 % (black). 100 % is corresponding to an activity of 1.5 U mg^{-1}

FMN (mM)	NADH (mM)		
	1.5	1	0.5
2	23	29	35
0.1	78	100	35
0.01	24	48	43
0.001	5	25	21
0	0	15	34

1.3. Conversion of D-xylose to D-xylonate using both NAD^+ regenerations systems

1.3.1. Eliminating the bias- pH, buffer, and temperature screening

To eliminate any biases when comparing both systems together a screening of pH, buffers, and temperature for both systems was performed simultaneously. Here, each system was coupled with *HsXyl*DH2 to convert D-xylose to D-xylonate in 100 µl scale at the different conditions: 4 buffers (HEPES, Bis-Tris, Tris-HCl and KPi), at 3 different pH (7, 7.5 and 8, for Bis-Tris: 6, 6.5 and 7) and 4 different temperatures (22, 30, 37 and 50 °C). 100 µl of each reaction were placed in the 96-well plate, the plate was sealed with gas-tight foil to eliminate evaporation. The D-xylonate was quantified in a 96-well plate using a spectrophotometric assay after Lien et al with some modifications, by measuring the absorbance at 550 nm after forming an hydroxymate complex.[1]

Each reaction was performed in replicas with 100 mM substrate and 1 mM NADH, the average yield was calculated and presented as a heat map taking the highest conversion value of the system as 100 %. To eliminate any possible artefacts or pipetting errors the reactions were repeated in replicas with the same conditions and were evaluated separately in the same way. Finally, the average of both runs was calculated and presented as a heat map taking the highest conversion value of each system as 100 % (Table 2).

Resonating with what was reported by Nowak et al,[2] the performance of NOX was at best at ambient temperatures between (22 and 30 °C). The effect of pH was mostly seen at lower temperatures with a clear preference towards more acidic pH and Bis-Tris as a buffer system. This effect was comprised at 30°C suggesting that the effect of temperature is more crucial for NOX. It is important to emphasize that all of these reactions were coupled with *HsXyl*DH2, unlike the previous study about enzyme characterization of NOX.

In the case of SH, as has been reported, the performance of SH was shown at best at pH 8 with a clear preference towards Tris-HCl and very poor performance with phosphate, most probably due to the possible presence of Na^+ resulting from titration. Na^+ ions are known to inhibit SH even at lower concentrations.[3] Interestingly, SH proved to be more suitable for operation at higher temperatures, where O_2 concentrations are low, with conversion yield reaching around half maximum at 50 C° and pH 8.

Concluding, the combination of Tris-HCl, pH 8, and 30 °C were the best conditions to compare both systems without any biases.

Supplementary Table 2: pH, buffer and temperatures screening of both SH and NOX coupled with HsXylDH2.

The conversion of D-xylose to D-xylonate was tested at different conditions. Equal amount of SH or NOX (1 µM) were added to 1 µM HsXylDH2 and 100 mM D-xylose, 1 mM NADH, for SH 0.1 mM of FMN were added. The amounts of D-xylonate were quantified by measuring the absorbance at 550 nm after forming the hydroxymate complex and presented as a heat map to the highest conversion in each system. Forming the hydromate was achieved after treating the samples with hydroxylamine and $FeCl_3$ according to Lien *et al.* [1]

*Bis-Tris was tested at pH 6, 6.5, and 7 in the table this corresponded to (7, 7.5, and 8 respectively). For all buffers the final concentration was 0.5 M, the temperature effect of the pH of the buffer was taken in consideration and the pH has been adjusted accordingly. Each reaction with the same conditions was conducted in independent replicates (n = 2, biologically independent). The mean values are represented in the table. SH: soluble hydrogenase, NOX: NADH oxidase.

System	run	Buffer	22 °C			30 °C			37 °C			50 °C		
			pH			pH			pH			pH		
			7	7.5	8	7	7.5	8	7	7.5	8	7	7.5	8
SH	1	HEPES	34	33	41	56	43	81	70	28	37	18	18	28
		Bis-Tris*	36	37	34	53	41	54	35	31	35	15	16	24
		Tris-HCl	47	43	42	43	100	99	46	41	38	20	22	35
		KPi	24	23	28	24	24	30	23	56	25	15	14	32
	2	HEPES	55	58	75	65	63	87	46	76	76	44	48	58
		Bis-Tris*	56	54	56	69	63	96	79	73	80	45	53	71
		Tris-HCl	57	67	61	77	82	100	79	87	69	60	60	59
		KPi	40	41	44	40	31	39	45	50	45	56	60	62
	average	HEPES	45	46	58	61	53	84	58	52	57	31	33	43
		Bis-Tris*	46	46	45	61	52	75	57	52	58	30	35	48
		Tris-HCl	52	55	52	60	91	100	63	64	54	40	41	47
		KPi	32	32	36	32	28	35	34	53	35	36	37	47

System	run	Buffer	22 °C			30 °C			37 °C			50 °C		
			pH			pH			pH			pH		
			7	7.5	8	7	7.5	8	7	7.5	8	7	7.5	8
NOX	1	HEPES	85	63	34	79	78	86	45	40	17	15	18	14
		Bis-Tris*	92	83	96	76	78	86	31	32	27	13	16	7
		Tris-HCl	82	50	74	64	100	93	12	12	11	12	11	9
		KPi	54	50	51	48	50	55	32	18	12	9	8	10
	2	HEPES	87	72	58	94	100	86	13	14	74	14	15	13
		Bis-Tris*	97	81	76	80	76	72	30	45	20	2	17	18
		Tris-HCl	58	33	79	74	75	95	9	10	14	11	10	11
		KPi	50	48	51	47	49	39	10	32	41	8	9	13
	average	HEPES	91	72	48	92	94	91	31	28	48	16	18	15
		Bis-Tris*	100	86	91	82	81	83	33	41	25	8	18	14
		Tris-HCl	74	44	81	73	93	99	12	12	14	13	12	11
		KPi	55	52	54	51	53	49	22	26	28	9	9	13

1.3.2. Influence of O_2, buffer capacity, and FMN

The effect of O_2 on the yield of biotransformation was examined. No significant difference was observed in samples with O_2. Despite that catalase was added to the reactions as a precaution.
Due to the expected high acidity resulting from the formation of D-xylonate, we tested the performance of both SH and NOX at a high buffer concentration of 400 mM. Increasing the buffer capacity had a significant impact on both systems. In the case of SH, the yield of D-xylonate increased by almost threefold (Table 3, entry 6). On the opposite with NOX samples, the yield of D-xylonate decreased by almost 50% (entry 8). Due to the high conversion of the substrate, the pH of the 100 mM buffer dropped, which might have affected the activity of SH more than the activity of this NOX variant, which has a pH optimum of 7 and is still stable at lower pH.[2] On the other hand, the pH optimum of SH is 8 and is stable at neutral pH.[3]
Furthermore, adding free FMN to the reaction proved to have a crucial impact on the yield of the biotransformation. FMN binds loosely to SH and gets lost during purification, FMN can be easily reconstituted into the purified SH by adding free FMN.[4]

Supplementary Table 3: The conversion of D-xylose to D-xylonate after 16 hours.

	Entry	Sample	D-xylonate (mM)	TTN (regeneration system)
a.	1	SH (anaerobic)	21.9 ± 3.5	1152
	2	SH	23.8 ± 2.8	1252
	3	SH (+ catalase)	24.8 ± 3.2	1305
	4	SH*(anaerobic)	30 ± 0.5	857
	5	NOX	33.9 ± 4.8	2266
b.	6	SH (anaerobic)	57.3 ± 0.5	3017
	7	SH (no FMN, anaerobic)	7.0 ± 0.7	368
	8	NOX	19.9 ± 0.3	1333

Reaction conditions: 500 µL of Tris-HCl buffer pH 8, containing (100 mM of D-xylose, 1 mM NADH and 0.1 mM FMN) in 2 mL tubes. For the anaerobic setup, the buffer was purged with pure N_2 for 15 min. The reaction was performed at 30 °C and shaking at 180 rpm. The concentration of the catalysts was 4 µM, 20 µM, and 15 µM for *HsXyl*DH2, SH, and NOX respectively. TTN is calculated as ŋ (product in µmol) / ŋ (regeneration system in µmol). SH: soluble hydrogenase, NOX: NADH oxidase, *HsXyl*DH2: xylose dehydrogenase.
a) 100 mM Tris-HCl. b) 400 mM Tris-HCl. *35 µM of SH.

1.4. Comparison between the open and closed system

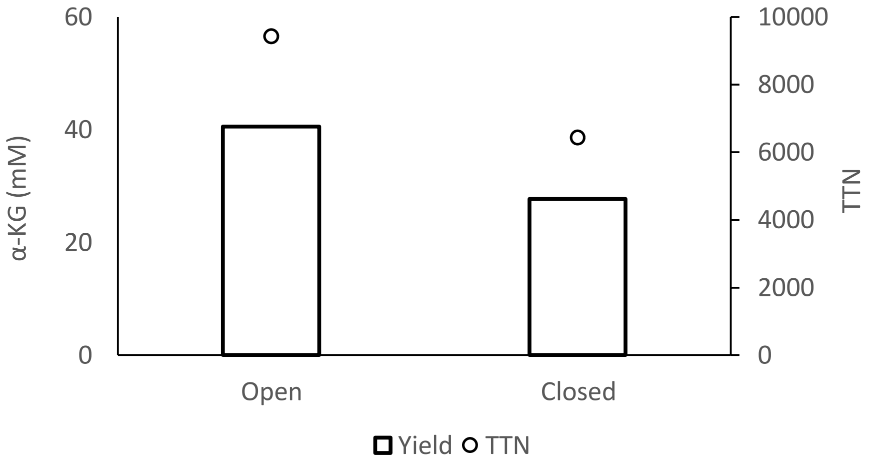

Supplementary Figure 2: The conversion of xylose to α-ketoglutarate (α-KG) in an open system with stirring and in a closed system. Reaction conditions: 100 mM D-xylose, 1 mM NADH, and 0.1 mM FMN at 30 °C, 400 mM Tris-HCl buffer, and 8.6 µM SH, all other enzymes were added according to their activity. The reaction in the open system was performed at 22 °C with stirring. Closed system: closed Eppendorf tubes. Open system: open glass vessel with stirring. TTN is highlighted by the circles. All other enzymes were added as stated in the experimental section.

1.5. The conversion of D-xylose to α-ketoglutarate overtime

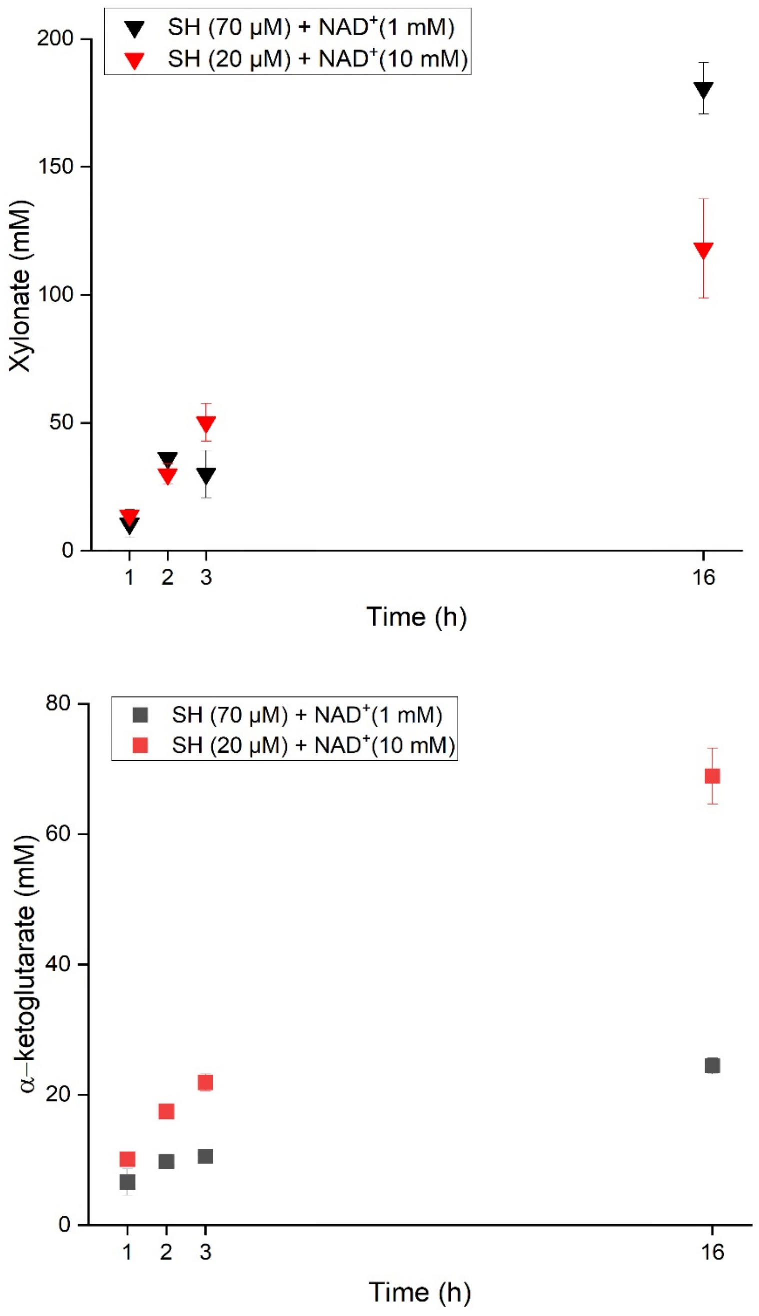

Supplementary Figure 3: The conversion of D-xylose to α-ketoglutarate (above) and the accumulation of D-xylonate (below) over time, at two different setups: 20 µM SH and 10 mM NADH (red) and 70 µM SH and 1 mM NADH (black). Reaction conditions: 500 µL of 1 M Tris-HCl pH 8 containing 0.5 M D-xylose, 1 mM $MgCl_2$, and 0.1 mM FMN in 10 mL deep well plates sealed with a breathable membrane. The reaction was conducted at 30 °C and 550 rpm. All other enzymes were added as stated in the experimental section. Each reaction with the same conditions was conducted in independent replicates (n = 2, biologically independent). The data are depicted as mean values with error bars as SD. SH: soluble hydrogenase.

1.6. Comparison between SH and NOX in a stirred setup

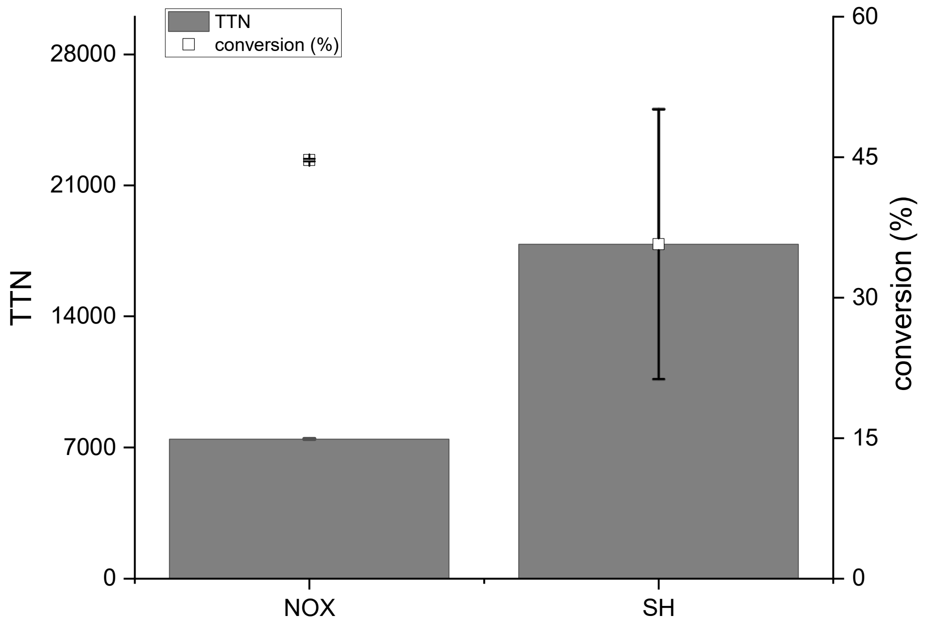

Supplementary Figure 4: The total turnover number of SH and NOX during the conversion of D-xylose to α-ketoglutarate at stirred setup. The productivity was calculated as the final total NAD^+ recycled in the system per enzyme unit during the whole reaction. The conversion yield is presented as white rectangulars and TTN as grey bars. The reaction was performed in 500 µL of 1 M Tris-HCl pH 8, with 0.15 M D-Xylose, 1 mM $MgCl_2$, 0.1 mM FMN,10 mM NADH at 22 °C, and stirring at 200 rpm using a magnetic stirrer for 16 hours. 0.32 mg of both enzymes were used, this equals approx.4 µM and 12 µM for SH and NOX respectively. All other enzymes were added as stated in the experimental section. Each reaction with the same conditions was conducted in independent replicates (n = 2, biologically independent). The high error bar in the SH sample is due to one sample where the H_2 senor was inserted, which affected the movement of the magnetic stirrer. The data are depicted as mean values with error bars as SD. SH: soluble hydrogenase, NOX: NADH oxidase.

1.7. H_2 production

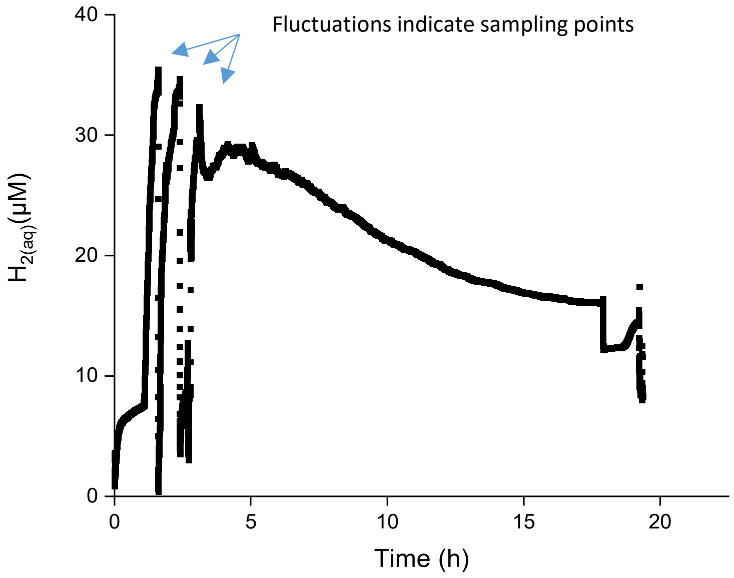

Supplementary Figure 5: The production of H_2 during the conversion of D-xylose to D-xylonate in a closed system over time. The reaction contained 100 mM D-xylose, 1 mM NADH, and 0.1 mM FMN and was purged with Ar. 15 µM of SH and HsXylDH2 were added at 22 °C. The H_2 evolution was measured using a microsensor dipped in solution in a septum-sealed vessel. 5 mM D-xylonate was produced.

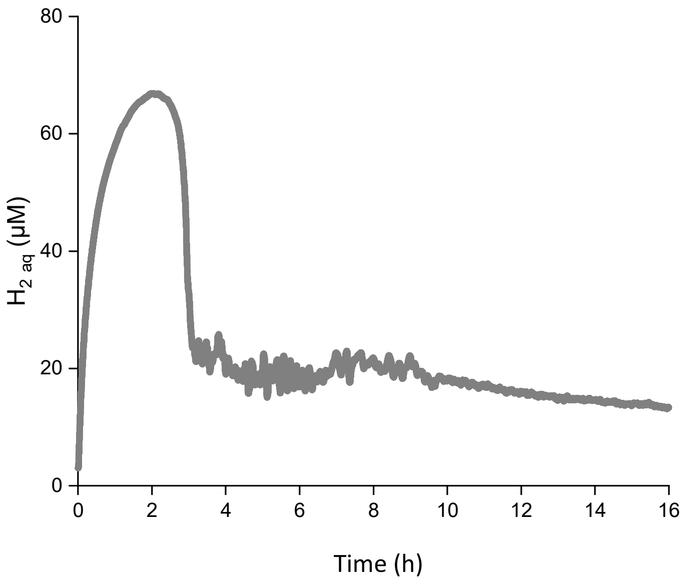

Supplementary Figure 6: The production of H_2 during the conversion of D-xylose to D-xylonate in the open system over time. The reaction contained 400 mM D-xylose, 1 mM NADH, and 0.1 mM FMN. Enzymes were added as 4 µM SH, 15 µM HsXylDH2, and 1 µM catalase at 22 °C. The H_2 evolution was measured using a microsensor dipped in solution in an open vessel.

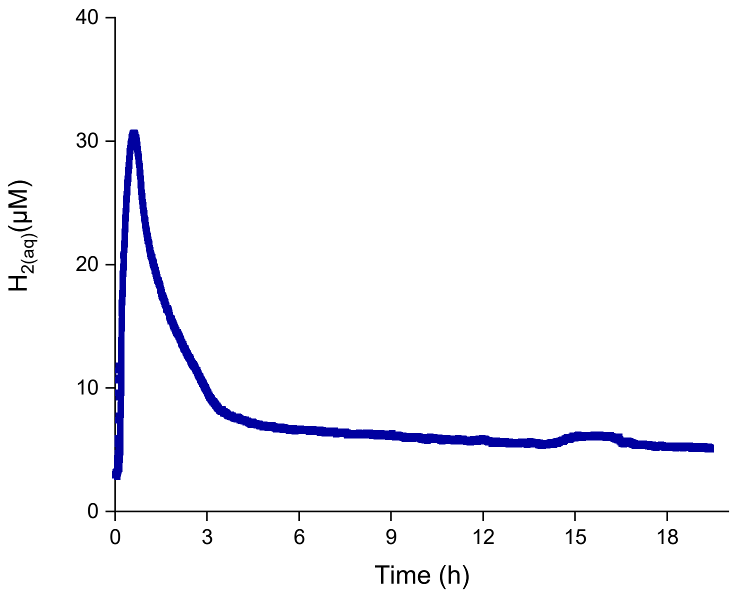

Supplementary Figure 7: The production of H_2 during the conversion of D-xylose to α-ketoglutarate in the open system with stirring over time.

Reaction conditions: 100 mM D-xylose, 1 mM NADH and 0.1 mM FMN, 400 mM Tris-HCl buffer, and 8.6 µM SH. The reaction in the open system was performed at 22 °C with stirring. All other enzymes were added as stated in the experimental section.

1.8. Diffusion of H_2 from the aqueous phase

We tested the diffusion of H_2 at 22°C using defined H_2 concentrations (figure 8S), as expected the diffusion rate increased by increasing the initial H_2 concentration. Determining the whole H_2 produced in the open system was extremely challenging due to the constant diffusion of H_2 into the gas phase measurement in the gas phase was also not accurate due to the dilution of H_2 in the air. We observed a continuous and rapid diffusion of H_2 into the gas phase at the reaction conditions (Figure 8). The diffusion rates changed depending on the H_2 concentration as expected. This prevented an accurate determination of the diffusion rate constant since H_2 was constantly produced by SH. A more delicate quantification of the released H_2 can be achieved in a continuous setup where a continuous flow of inert gas removes H_2 from the system and measures it at the exhaust. A similar setup was used to quantify the H_2 produced from D-xylose in the gas phase.[5] It is also worth mentioning that a part of H_2 is oxidized during the measurement by the sensor. The only way that could prevent a full conversion of substrate to H_2 is the O_2 reduction to H_2O_2, H_2O, and superoxide as has been reported.[6] Despite that we could not experimentally determine the full conversion of substrate to H_2 due to the above-mentioned technical challenges. We still think that the majority of the substrate was indeed converted to H_2 for two reasons: first, the data from NOX showed a limited O_2 availability in the solution even in the open system. In addition, the highly produced H_2 will push the remained O_2 out of the solution. Second, O_2 reduction was observed in a solution with 40% of O_2 saturation which is as twice the initial concentration of O_2 in all of our reactions (ambient concentration). Furthermore, samples without catalase showed no detectable amounts of H_2O_2. All of this supports the fact that O_2 reduction was not that significant to lower the H_2 yield.

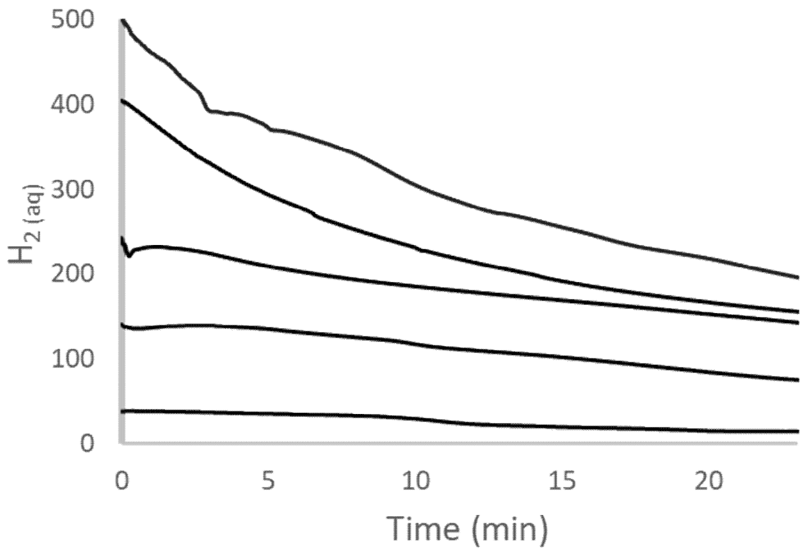

Supplementary Figure 8: The diffusion of H_2 from buffer solution (1 M Tris-HCl, pH 8) at 22 °C. Defined H_2 concentrations were aliquoted into open vessels. The concentration of the dissolved H_2 was measured over time. The diffusion rates were calculated depending on each initial H_2 concentration.

1.9. Heterologous production of SH in *E. coli*.

Supplementary Table 4: Comparison of the yields and specific activities of SH expressed in different hosts.

Host	Production [mg L^{-1} culture]	NADH oxidation activity [U mg^{-1}]	Production [U L^{-1} day^{-1}]	Reference
E. coli [a]	9.1-12.3	1.2	10.7	This study
R. eutropha [b]	7.5-22.3	1.5	1.86	This study and 6
E. coli [c]	4.5	Not determined	3.3 [d]	7

All data correspond to highly purified proteins.
[a] 1 day of cultivation and protein expression
[b] 8 days of cultivation and protein expression
[c] 2 days of cultivation and protein expression
[d] Assuming the same activity as in *R. eutropha* for NADH oxidation. Only the H_2-driven NAD^+ activity was measured here as 7.2 U mg^{-1} similar to in *R. eutropha.*

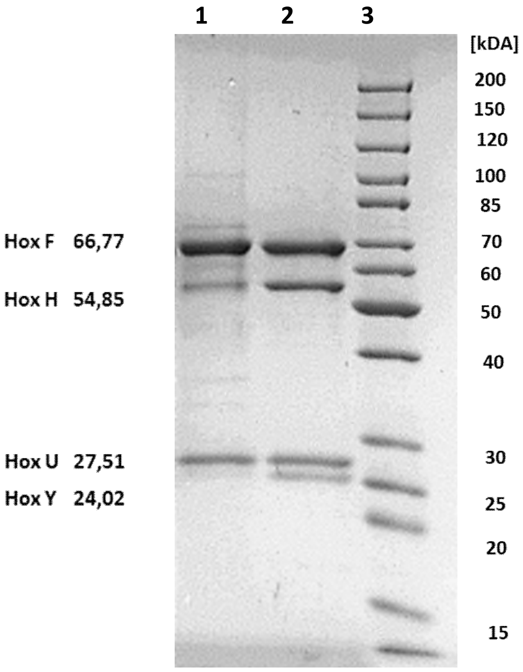

Supplementary Figure 9: SDS-PAGE analyses of the SH. SH was produced in *E. coli* lane 1, in *R. eutropha* lane 2, and the marker (PageRuler™, unstained, Thermo Scientific) lane 3. The SH purification was performed multiple times from independent cultivations, the same SDS-PAGE profile was observed. The SDS-PAGE gels of all independent samples are shown in Source data file.

1.10. Scaling up.

Supplementary Table 5: The conversion of D-xylose to α-ketoglutarate after 44 hours in scaled-up setups.

Entry	Volume (mL)	Stirring (rpm)	SH (µM)	α-KG (mM)	TTN
1 [a]	9	60	7	27	8000
2 [b]	10	60	7	37	9143
3 [c]	40	0	1	10.8	17000
4 [d]	50	200	3	9.1	2733

Reaction conditions: 1M Tris-HCl pH 8 containing 0.1 M D-xylose, 1 mM $MgCl_2$, 10 mM $NAD^{+,}$ and 0.1 mM FMN at 22 °C the reaction was degassed with N_2 before adding the enzymes. All other enzymes were added as stated in the experimental section. The reaction was performed under a constant flow of N_2 (2.8-2.2 mL min^{-1}) in a glass flask with a gas inlet and outlet. The gas at the exhaust was analyzed *via* GC-TCD to determine the amount of H_2.

[a] 10 mM NADH instead of NAD^+. 10 mL but 1 mL was lost during degassing.
[b] 70 % of SH was added at the beginning goof the reaction and the rest 30 % after 18 hours.
[c] After 5 days, 10 mM NADH instead of NAD^+. 1/10 of all other enzymes were added compared to standard concentrations.
[d] Yield obtained after 18 hours without further increase. 1/10 of all other enzymes were added compared to standard concentrations.

2. Supplementary References

1. Lien OG. Determination of Gluconolactone, Galactonolactone, and Their Free Acids by Hydroxamate Method. *Analytical Chemistry* **31**, 1363-1366 (1959).

2. Nowak C, Beer B, Pick A, Roth T, Lommes P, Sieber V. A water-forming NADH oxidase from Lactobacillus pentosus suitable for the regeneration of synthetic biomimetic cofactors. *Frontiers in Microbiology* **6**, (2015).

3. Ratzka J, Lauterbach L, Lenz O, Ansorge-Schumacher MB. Systematic Evaluation of the Dihydrogen-Oxidising and NAD^+-Reducing Soluble [Nife]-Hydrogenase from *Ralstonia Eutropha* H16 as a Cofactor Regeneration Catalyst. *Biocatalysis and Biotransformation* **29**, 246-252 (2011).

4. van der Linden E*, et al.* Selective release and function of one of the two FMN groups in the cytoplasmic NAD+-reducing [NiFe]-hydrogenase from *Ralstonia eutropha*. *European Journal of Biochemistry* **271**, 801-808 (2004).

5. Martín del Campo JS*, et al.* High-Yield Production of Dihydrogen from Xylose by Using a Synthetic Enzyme Cascade in a Cell-Free System. *Angewandte Chemie International Edition* **52**, 4587-4590 (2013).

6. Lauterbach L, Lenz O. Catalytic Production of Hydrogen Peroxide and Water by Oxygen-Tolerant [NiFe]-Hydrogenase during H_2 Cycling in the Presence of O_2. *J Am Chem Soc* **135**, 17897-17905 (2013).

7. Schiffels J, Pinkenburg O, Schelden M, Aboulnaga E-HAA, Baumann MEM, Selmer T. An innovative cloning platform enables large-scale production and maturation of an oxygen-tolerant [NiFe]-hydrogenase from *Cupriavidus necator* in *Escherichia coli*. *PloS one* **8**, e68812-e68812 (2013).
